# Leukotriene B4 receptor-2 contributes to KRAS-driven lung tumor formation by promoting interleukin-6-mediated inflammation

**DOI:** 10.1038/s12276-021-00682-z

**Published:** 2021-10-11

**Authors:** Jae-Hyun Jang, Donghwan Park, Guen-soo Park, Dong-Wook Kwak, JaeIn Park, Dae-Yeul Yu, Hye Jin You, Jae-Hong Kim

**Affiliations:** 1 Department of Biotechnology, College of Life Sciences and Biotechnology, Korea University, Seoul, 02841 Republic of Korea; 2 Disease Model Research Laboratory, Genome Editing Research Center, Korea Research Institute of Bioscience and Biotechnology (KRIBB), Daejeon, 34141 Republic of Korea; 3 Division of Translational Science, National Cancer Center, Goyang, 10408 Republic of Korea; 4 Division of Life Sciences, College of Life Sciences, Korea University, Seoul, 02841 Republic of Korea

**Keywords:** Non-small-cell lung cancer, Lipid signalling

## Abstract

Although lung cancer is the leading cause of cancer-related deaths worldwide and KRAS is the most frequently mutated oncogene in lung cancer cases, the mechanism by which KRAS mutation drives lung cancer has not been fully elucidated. Here, we report that the expression levels of leukotriene B_4_ receptor-2 (BLT2) and its ligand-producing enzymes (5-LOX, 12-LOX) were highly increased by mutant KRAS and that BLT2 or 5-/12-LOX blockade attenuated KRAS-driven lung cell proliferation and production of interleukin-6 (IL-6), a principal proinflammatory mediator of lung cancer development. Next, we explored the roles of BLT2 and 5-/12-LOX in transgenic mice with lung-specific expression of mutant KRAS (Kras^G12D^) and observed that BLT2 or 5-/12-LOX inhibition decreased IL-6 production and tumor formation. To further determine whether BLT2 is involved in KRAS-driven lung tumor formation, we established a Kras^G12D^/BLT2-KO double-mutant mouse model. In the double-mutant mice, we observed significantly suppressed IL-6 production and lung tumor formation. Additionally, we observed high BLT2 expression in tissue samples from patients with KrasG12D-expressing lung adenocarcinoma, supporting the contributory role of BLT2 in KRAS-driven human lung cancer. Collectively, our results suggest that BLT2 is a potential contributor to KRAS-driven lung cancer and identify an attractive therapeutic target for KRAS-driven lung cancer.

## Lung cancer: A strategy for sidestepping a menacing mutation

A protein that fuels inflammation could offer a therapeutic target to allow clinicians to bypass a highly tumorigenic but hard-to-treat mutant protein found in many lung cancers. Researchers have long struggled to develop medicines that inhibit KRAS, a signaling protein that commonly acquires mutations that turn it into a potent driver of cancerous growth. As an alternative, researchers led by Jae-Hong Kim of Korea University, Seoul, set out to identify proteins that govern the tumorigenic inflammatory response initiated by KRAS. They showed that a protein called BLT2 is highly expressed in KRAS-mutant lung cancer cells, and appears to contribute strongly to KRAS-mediated inflammation and tumor growth in mouse models. BLT2 could thus offer a useful downstream target for blocking the impact of mutant KRAS without grappling with the challenges of directly inhibiting KRAS.

## Introduction

Lung cancer is the leading cause of cancer-related deaths among both men and women, with more than 1.5 million deaths per year worldwide^1^. Epidermal growth factor receptor (EGFR) mutations are the second most common cause of lung cancer, and KRAS is the most frequently mutated oncogene in lung cancer cases^2^. Despite several notable studies^3,4^, mutant KRAS is difficult to target with small molecules due to its undruggable structure^5^. Because of these limitations, alternative approaches involving lung inflammatory pathways have been explored^6^. Interleukin-6 (IL-6) is a proinflammatory cytokine that has been implicated in the progression of lung cancers, including KRAS-driven lung cancer^7^. IL-6 contributes to lung cancer progression by triggering pro-inflammatory pathways that enhance cancer cell proliferation; thus, IL-6 is considered a marker of poor prognosis in lung cancer^8–10^. Unfortunately, IL-6 blockade has limited effectiveness in lung cancer patients^11,12^, and therefore, alternative molecular targets are required.

Leukotriene B_4_ (LTB_4_) is a potent proinflammatory lipid mediator that binds to the cell surface G protein-coupled receptors LTB_4_ receptor-1 (BLT1) and BLT2^13^. BLT1 is expressed mainly on peripheral leukocytes, while BLT2 is minimally expressed under normal conditions but overexpressed in proinflammatory environments^14–16^. Moreover, recent reports have indicated that the BLT2 signaling cascade is associated with lung inflammatory diseases such as asthma^17,18^. Additionally, BLT2 and its ligand-producing enzymes 5-lipoxygenase (5-LOX) and 12-lipoxygenase (12-LOX), which convert arachidonic acid into the BLT2 ligands LTB_4_ and 12(*S*)-hydroxy-5Z,8Z,10E,14Z-eicosatetraenoic acid (12(*S*)-HETE), respectively^19,20^, play critical roles in the production of proinflammatory cytokines in lung airway disease in the context of asthma^21^. Despite the suggested role of the BLT2 cascade in lung inflammation, little is known about its contribution to the progression of lung cancer, especially KRAS-mutant lung cancer.

To this end, in this study, we investigated whether BLT2 contributes to mutant KRAS-driven lung cancer progression. We found that the BLT2 cascade lies downstream of mutant KRAS and contributes to mutant KRAS-driven lung cancer cell proliferation and IL-6 production. Additionally, we revealed that the inhibition of BLT2 decreases lung tumor formation, IL-6 production, and inflammatory phenotypes in the lung. Furthermore, *Blt2* knockout (KO) in a mouse model of KRAS-driven lung cancer was associated with strong suppression of lung tumor formation and IL-6 production. Moreover, immunohistochemical (IHC) and immunofluorescence (IF) analysis revealed substantial BLT2 expression in tissue samples from lung adenocarcinoma patients with the KrasG12D mutation. Thus, we identified BLT2 as a potential contributor to KRAS-driven lung cancer, and our results may facilitate the development of strategies against KRAS-mutant lung cancer.

## Materials and methods

### Chemicals and plasmids

RPMI 1640 medium and DMEM were purchased from Corning, Inc. (Corning, NY, USA). Fetal bovine serum (FBS) was purchased from HyClone Laboratories, Inc. (Logan, UT, USA). MK886, baicalein, and LY255283 were purchased from Cayman Chemical (Ann Arbor, MI, USA). SB203580 was purchased from Calbiochem (San Diego, CA, USA). Dimethyl sulfoxide (DMSO) was purchased from Sigma-Aldrich (St. Louis, MO, USA). The pCGN vectors were purchased from Invitrogen (Carlsbad, CA, USA). Antibodies against BLT2 were obtained from Enzo Life Sciences, Inc. (Farmingdale, NY, USA); antibodies against 5-LOX, β-actin, phosphorylated cytosolic phospholipase A_2_ (p-cPLA_2_), and p-IκBα were obtained from Cell Signaling Technology (Danvers, MA, USA), and antibodies against cPLA_2_ and 12-LOX were obtained from Santa Cruz Biotechnology, Inc. (Dallas, TX, USA). All other chemicals were obtained from standard sources and were of molecular biology grade or higher.

### Cell culture and transfection

BEAS-2B, A549, and SK-LU-1 cells were obtained from the Korean Cell Line Bank (KCLB, 10061). A549 and SK-LU-1 cells were grown in DMEM supplemented with 10% FBS and antibiotic–antimycotic solution (Gibco; El Paso, TX, USA) at 37 °C in a 5% CO_2_ humidified atmosphere. BEAS-2B cells were grown in an RPMI medium under the same conditions. For transient transfection, cells were seeded at 2 × 10^5^ cells per 60-mm plate and incubated for 24 h. Then, DNA–lipid complexes were formed with 4 μl of Lipofectamine (Invitrogen) and 2 μg of DNA and added to each dish for 18 h. Empty vector DNA was used to equalize the total quantities of transfected DNA between each experiment.

### RNA interference (siRNA)

KRAS-specific and control (scrambled) siRNAs were purchased from Bioneer (Daejeon, Korea). The siRNAs were introduced into cells by transfection as described above for 18 h in Opti-MEM solution (Invitrogen) using Oligofectamine reagents (Invitrogen).

### Reverse transcription (RT)-PCR

Total RNA was extracted from cells or mouse lung tissue using the easy-BLUE^TM^ Total RNA Extraction Kit (Intron, Gyeonggi-do, Korea). Two micrograms of RNA were subjected to RT with M-MLV reverse transcriptase (Beams Bio, Gyeonggi-do, Korea) followed by semiquantitative PCR with a PCR PreMix Kit (Intron). The primer sequences are provided in Supplementary Table 1. The final PCR products were resolved on a 1.5% agarose gel containing ethidium bromide and visualized under UV light.

### Western blotting

Cells were collected and lysed with buffer containing 40 mM Tris–HCl (pH 8.0), 120 mM NaCl, 0.1% Nonidet-P40, 100 mM phenylmethylsulfonyl fluoride, 1 mM sodium orthovanadate, 2 g/ml leupeptin, 2 g/ml aprotinin, and phosSTOP cocktail (Roche, Basal, Switzerland). Mouse lung tissues were homogenized with buffer containing 100 mM Tris–HCl (pH 7.4), 150 mM NaCl, 1 mM EGTA, 1 mM EDTA, 1% Triton X-100, 0.5% sodium deoxycholate, and proteinase inhibitor cocktail. Proteins were separated by sodium dodecyl sulfate–polyacrylamide gel electrophoresis and transferred to a polyvinylidene fluoride membrane. The membrane was blocked with 5% nonfat dry milk in Tris-buffered saline for 1 h and incubated with primary antibodies for 1 h at room temperature. The membrane was washed for 1 h, and blots were incubated with a peroxidase-conjugated secondary antibody for 1 h and then washed for 1 h. The bands were visualized by enhanced chemiluminescence (Amersham Biosciences, UK).

### Cell counting

Cells were plated at a density of 5.0 × 10^4^ cells per well in 24-well plates and incubated in medium containing 10% FBS for 24 h. Then, the cells were incubated with the indicated inhibitors or DMSO in a medium containing 2% FBS for 24 or 48 h. For measurement of cell proliferation, cells were trypsinized and counted with a hemocytometer using the trypan blue exclusion method.

### Mouse models and animal care

Kras^G12D^ mice were kindly provided by Professor Dae-Yeol Yu, who generated these mice via DNA microinjection into B6D2F1 strain (BDF-1) mouse embryos as previously described^22^; a KrasG12D gene expression vector containing the hSP-C promoter was used to drive lung-specific expression of the KrasG12D gene. BLT2 KO mice were constructed as previously described^23^. The mouse room was maintained at a temperature of 22 ± 1 °C and a humidity of 50 ± 10% with a 12-h light/dark cycle. All animal procedures were conducted in accordance with the guidelines of the Korea University Institutional Animal Care & Use Committee (KUIACUC 2019-0056).

### Inhibitor treatment of the KrasG12D mutant mice

Beginning at 9 weeks of age, the Kras^G12D^ mice and control littermates were injected intraperitoneally (i.p.) with 0.5 mg/kg MK886, 5 mg/kg baicalein, or 2.5 mg/kg LY255283 once weekly for 12 weeks. At the experimental endpoint (21 weeks of age), five mice from each group were sacrificed and used for analysis. No experimental mice were excluded from any of these analyses.

### Generation of Kras^G12D^/BLT2 KO mice

For the construction of the Kras^G12D^/BLT2 KO mouse model, *Blt2* KO mice on the C57BL/6 background were mated with wild-type (WT) BDF-1 mice to at least the F7 generation. After the acquisition of the BDF-1 strain of *Blt2* KO mice, Kras^G12D^/BLT2 KO double-mutant mice were generated by crossing KrasG12D mice with BLT2 KO mice (Supplementary Fig. 1). We used the primers shown in Supplementary Table 1 for genotyping.

### Lung histology and analysis of cells in bronchoalveolar lavage fluid (BALF)

Lung section (5 μm thickness) were mounted on Superfrost Plus glass slides (Fisher Scientific, Pittsburgh, PA, USA), deparaffinized, and stained with hematoxylin and eosin (H&E). Inflammatory cells in BALF were collected by centrifugation (1000 × *g* for 3 min) and washed with PBS. Next, BALF cells were fixed on glass slides and stained with H&E. Images were acquired using a BX51 microscope (Olympus, Tokyo, Japan) equipped with a DP71 digital camera (Olympus).

### Enzyme-linked immunosorbent assay (ELISA)

Human LTB_4_ and 12(*S*)-HETE levels were measured using commercially available ELISA kits (Enzo Life Sciences, Inc.), and IL-6 levels were measured using a commercially available ELISA kit (BD Biosciences; Bedford, MA, USA). The LTB_4_, 12(*S*)-HETE, and IL-6 concentrations were measured according to the manufacturers’ instructions.

### IHC and IF analyses

Paraffin-embedded lung tissues from lung adenocarcinoma patients were purchased from US Biomax (Derwood, MD, USA) and subjected to IHC and IF analyses. The patient samples were deparaffinized, and antigen retrieval was performed by microwaving the sample in a 0.01 M sodium citrate buffer. The samples were incubated with primary antibodies against KrasG12D (ab221163, Abcam, Cambridge, UK; 7.9 μg/ml) and BLT2 (PA533911, Thermo Scientific, Waltham, MA, USA; 12 μg/ml). Endogenous peroxidase activity was blocked with 0.3% H_2_O_2_. Samples were stained using the avidin–biotin–peroxidase complex method with an ABC staining kit (Thermo Scientific) and then counterstained with hematoxylin. The stained sections of the lung were observed using a BX51 microscope (Olympus, Tokyo, Japan) equipped with a DP71 digital camera (Olympus). For IF staining of KrasG12D and BLT2, primary antibodies against KrasG12D (ab221163, Abcam) and BLT2 (ADI-905-794-100, Enzo Life Sciences, Farmingdale, NY, USA) were conjugated with FITC and PE, respectively, using a conjugation kit (ab188285, ab102918, Abcam). The patient samples were deparaffinized, rehydrated, and blocked with buffer (PBS containing 1% BSA) for 1 h at RT. The lung tissues were then incubated overnight at 4 °C with FITC-conjugated KrasG12D and PE-conjugated BLT2 antibodies. After three washes in PBS, the slides were incubated with DAPI (Sigma-Aldrich, St. Louis, MO, USA). The slides were washed in PBS, mounted, and observed under a confocal laser scanning microscope (LSM 700, Carl Zeiss, Oberkochen, Germany). Specific information on the patient-derived lung adenocarcinoma tissues is provided in Table 1.

**Table 1:** Information on the lung tissues used in the study.

	Age	Sex	Organ/anatomic site	Pathological diagnosis	Grade	Type	KrasG12D-positive area (%)	BLT2-positive area (%)
Fig. 5a	47	Male	Lung	Normal			0	0
Fig. 5b	68	Male	Lung	Adenocarcinoma	2	Malignant	8.1	7.5
Fig. 5c	72	Male	Lung	Adenocarcinoma	2	Malignant	16.1	15.9
Fig. 5d (left)	35	Male	Lung	Normal			0	0
Fig. 5d (right)	55	Male	Lung	Adenocarcinoma	2	Malignant	9.2	8.1

### Comparison of BLT2 gene amplification in the KRAS mutant and KRAS wild type samples

For analysis of BLT2 gene amplification in KRAS mutant lung cancer patients, we analyzed the Pan-Lung Cancer database (TCGA, Nat Genet 2016) cohort of 1144 lung cancer patients via cBioPortal based on the KRAS mutation and BLT2 gene amplification (http://www.cbioportal.org/study/summary?id=nsclc_tcga_broad_2016).

### Statistical analysis

All quantitative data are shown as the mean ± SD of three independent experiments. Statistical comparisons between experimental groups were performed using Student’s *t*-test. *p* < 0.05 was considered significant. *p* < 0.05, *p* < 0.01, and *p* < 0.001 are designated by *, **, and ***, respectively.

## Results

### BLT2, 5-LOX, and 12-LOX expression is significantly increased in KRAS-mutant lung cancer cells

**Fig. 1:**
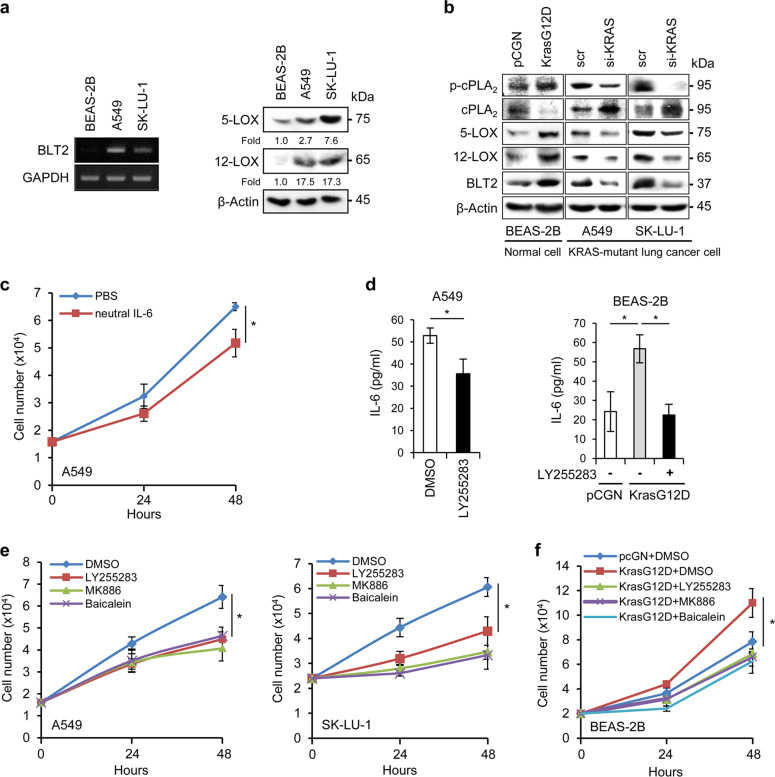
The BLT2 cascade lies downstream of mutant KRAS in lung cancer cells. **a** *BLT2* mRNA expression and 5-/12-LOX protein expression in BEAS-2B normal lung cells and in A549 and SK-LU-1 KRAS-mutant lung cancer cells. The results are representative of three independent experiments with similar results. **b** Levels of BLT2 cascade proteins in cell lysates of the indicated cell lines. KrasG12D overexpression was induced in BEAS-2B cells by transfection with a KrasG12D expression plasmid. KRAS was knocked down in A549 and SK-LU-1 cells using siRNA-KRAS. Scr scrambled siRNA control. The western blot results are representative of three independent experiments with similar results. Band intensities were quantified using ImageJ and are expressed as the fold change relative to the control value. **c** A549 cells were treated with an anti-IL-6 neutralizing antibody (100 ng/ml). Cells were then counted with a hemocytometer. The data are presented as the mean ± SD values of three independent experiments. **d** ELISAs of IL-6 production in cell culture supernatants. A549 cells (left) were treated with the BLT2-specific inhibitor LY255283 (10 μM). BEAS-2B cells (right) were transfected with the KrasG12D expression plasmid. Twenty-four hours after transfection, the cells were exposed to LY255283 (10 μM). The data are presented as the mean ± SD values of three independent experiments. **e** A549 (left) and SK-LU-1 cells (right) were treated with inhibitors of BLT2, 5-LOX, and 12-LOX (10 μM LY255283, 5 μM MK886, and 800 nM baicalein, respectively) and then counted. The data are presented as the mean ± SD values of three independent experiments. **f** BEAS-2B cells were transfected with the KrasG12D expression plasmid. Twenty-four hours after transfection, the cells were exposed to the indicated inhibitor and then counted. Cell proliferation data were statistically analyzed at 48 h and compared between the indicated inhibitor groups and the DMSO control group. The data are presented as the mean ± SD values of three independent experiments. The data were analyzed using an unpaired two-tailed Student’s *t*-test. *p < 0.05.

We began our study of the role of BLT2 in KRAS-mutant lung cancer by examining the basal expression levels of BLT2, 5-LOX, and 12-LOX in KRAS-mutant lung cancer cells. The expression levels of BLT2, 5-LOX, and 12-LOX were increased (Fig. 1a) in two human lung cancer cell lines with activating KRAS mutations (A549 with the KrasG12S mutation, and SK-LU-1 with the KrasG12D mutation) compared with a noncancerous control cell line (BEAS-2B, human bronchial epithelial cell line).

To determine whether mutant KRAS expression is associated with the expression of the BLT2 cascade, we transfected BEAS-2B cells with a mutant KRAS expression plasmid (pCGN-Kras^G12D^). The KrasG12D-expressing BEAS-2B cells showed increased protein levels of 5-LOX, 12-LOX, and BLT2 compared with the control vector-expressing BEAS-2B cells (Fig. 1b). Similarly, the levels of phosphorylated cPLA_2_ (p-cPLA_2_), an activated form of cPLA_2_ that catalyzes the production of arachidonic acid, a precursor molecule for eicosanoid synthesis^24^, were strongly increased by mutant KRAS expression (Fig. 1b). In addition, we used a targeted small interfering RNA to knock down KRAS (si-KRAS) in A549 and SK-LU-1 cells and found that the levels of p-cPLA_2_, 5-LOX, 12-LOX, and BLT2 were substantially reduced compared with those of the scrambled siRNA control (Fig. 1b). Collectively, these results suggest that mutant KRAS regulates the expression of the BLT2 cascade in lung cancer cells.

### BLT2 or 5-/12-LOX inhibition decreases IL-6 production and cell proliferation

Recent studies have demonstrated that IL-6 is critical for lung tumor progression and thus acts as a key driver of lung cancer^25–27^. Accordingly, we observed that treatment of A549 lung cancer cells with an anti-IL-6 neutralizing antibody significantly reduced cell proliferation, as indicated by the 20.4% decrease in the number of cells in the cell count assays (Fig. 1c), suggesting that IL-6 production is associated with KRAS-mutant lung cancer cell proliferation. Next, to determine whether BLT2 mediates IL-6 production, we evaluated the effect of treatment with a BLT2-specific antagonist (LY255283) on IL-6 levels in A549 cell culture supernatants by ELISAs; this treatment strongly reduced the IL-6 levels (Fig. 1d, left panel). Additionally, the levels of IL-6 in the BEAS-2B cells transfected with the KrasG12D expression plasmid showed a 2.3-fold increase, but this enhancement was almost completely abrogated by treatment with LY255283 (Fig. 1d, right panel). These results suggest that BLT2 mediates IL-6 production in KRAS-mutant lung cancer cells.

To determine whether inhibition of the BLT2 cascade affects the proliferation of A549 and SK-LU-1 cells, we treated cells with MK886 to inhibit 5-LOX^28,29^ or baicalein to inhibit 12-LOX^30,31^. Inhibition of 5-LOX, 12-LOX or BLT2 clearly suppressed the proliferation of A549 and SK-LU-1 cells (Fig. 1e). Similarly, when BEAS-2B cells were transfected with the KrasG12D expression plasmid, cell proliferation was strongly enhanced, but this enhancement was almost completely attenuated by inhibition of 5-/12-LOX or BLT2 (Fig. 1f).

### Increased expression of BLT2 cascade molecules in a mouse model of KrasG12D-driven lung cancer

**Fig. 2:**
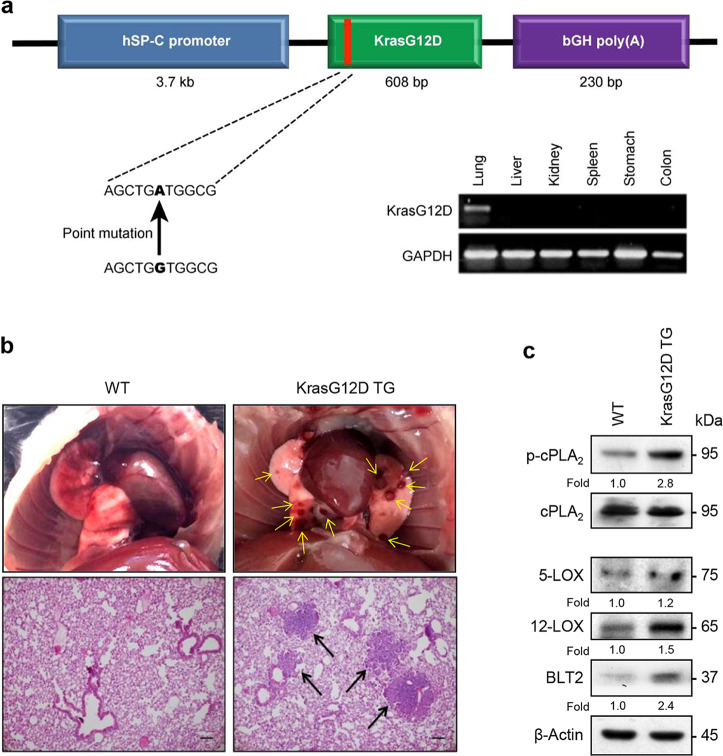
The levels of 5-/12-LOX and BLT2 are increased in the Kras^G12D^ mice. **a** Strategy with the promoter and transgene was used to establish lung-specific KrasG12D expression in mice. **b** Representative images of lung lesions and H&E-stained lung sections from the indicated groups of 8-week-old mice. The arrows indicate lung tumors. Scale bars, 50 μm. **c** The protein levels of p-cPLA_2_, 5-/12-LOX, and BLT2 in the lung tissue extracts of the Kras^G12D^ mice were analyzed by western blotting. Total cPLA_2_ and β-actin were used as controls. Three mice were tested for analysis. Similar results were obtained, and representative data are shown. Band intensities were quantified using ImageJ and are expressed as a fold of the control value (cPLA_2_ as a control for p-cPLA_2_; β-actin as a control for 5-LOX, 12-LOX, and BLT2).

To examine the effect of BLT2 signaling on nodule progression in KRAS-mutant lung cancer in vivo, we used a transgenic mouse model with a lung-specific expression of mutant KRAS (Kras^G12D^ mice). In this model, mutant KrasG12D expression is driven by the promoter of human surfactant protein C (hSP-C) (Fig. 2a, top), which is expressed only in the lung (Fig. 2a, bottom right). In the Kras^G12D^ mice, nodules were observed only in the lungs from 8 weeks after birth (Fig. 2b). Then, we assessed the basal levels of p-cPLA_2_, 5-LOX, 12-LOX, and BLT2 in lung tissue lysates. As shown in Fig. 2c, the levels of p-cPLA_2_, 5-LOX, 12-LOX, and BLT2 were strongly enhanced in the lung tissues of the Kras^G12D^ mice compared with those of the WT mice. These results suggest that the expression of members of the p-cPLA_2_-5-/12-LOX-BLT2 cascade is upregulated in the KrasG12D-driven lung cancer mouse model.

### Inhibition of 5-/12-LOX or BLT2 suppresses IL-6 production and KrasG12D-driven lung nodule formation

**Fig. 3:**
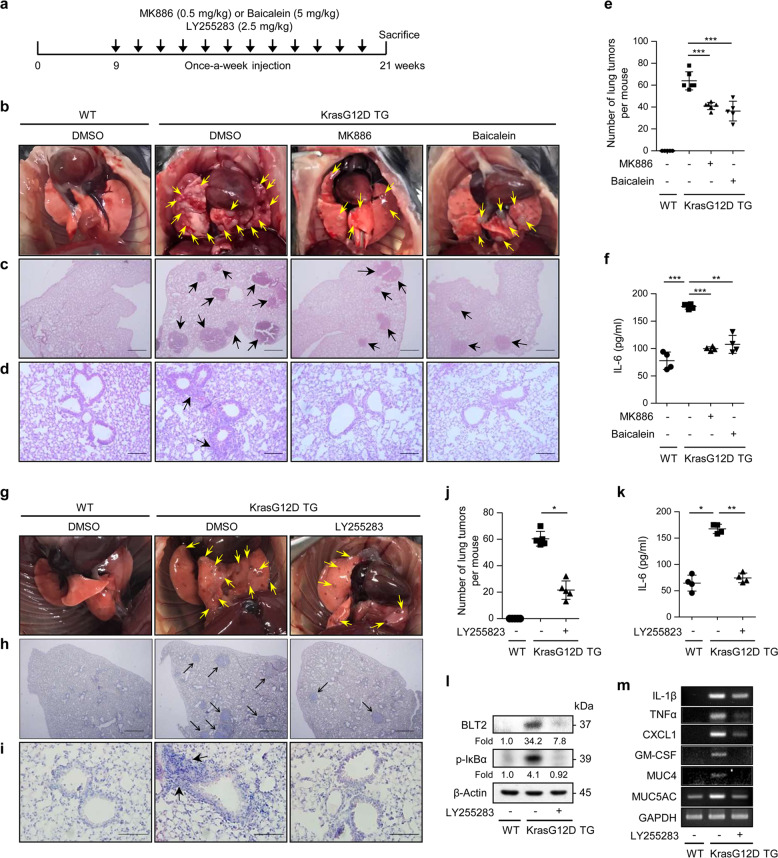
Inhibition of the 5-/12-LOX-BLT2 cascade suppresses KrasG12D-driven lung tumor formation and IL-6 production in the Kras^G12D^ mice. **a** Experimental schedule for BLT2 cascade inhibition in the Kras^G12D^ mice. **b**, **g** Representative images of lung lesions. **c**, **h** H&E staining of lung tissues. Scale bars, 500 μm. The arrows indicate lung tumors. **d**, **i** Airway cellular infiltration in the indicated experimental groups. Scale bars, 100 and 200 μm, respectively. The arrows indicate infiltrated cells. **e**, **j** The number of lung tumors in each mouse was counted. The data are presented as the mean ± SD; *n* = 5. *p < 0.05 and ***p < 0.001 according to Student’s *t*-test. **f**, **k** Lung tissue extract was collected for IL-6 measurement by ELISAs. The data are shown as the mean ± SD; *n* = 5. *p < 0.05, **p < 0.01 and ***p < 0.001 according to Student’s *t*-test. **l** The expression level of BLT2 and phosphorylation level of IκBα in lung tissue extracts were assessed by western blotting. The results are representative of *n* = 3 independent experiments. Band intensities were quantified using ImageJ and are expressed as the fold change relative to the control value. **m** Total RNA was isolated from mouse lungs, and semiquantitative PCR analysis of the indicated mRNAs was conducted. The data are representative of three independent experiments with similar results.

Lung nodules were observed beginning at 8 weeks after birth in the Kras^G12D^ mice. To examine the suppressive effects of BLT2 cascade inhibition on KrasG12D-driven lung nodule formation, we injected inhibitors into the peritoneal cavity of the mice starting 9 weeks after birth once a week for 12 weeks (Fig. 3a). Interestingly, the number of lung nodules was significantly decreased by 5- or 12-LOX inhibitor treatment (Fig. 3b, c, e), and the elevated IL-6 production in lung tissue was almost completely suppressed (Fig. 3f). We also investigated whether BLT2 inhibition could suppress tumor formation and IL-6 production in these mice and observed that treatment with LY255283 clearly suppressed KRAS-driven lung tumor formation (Fig. 3g–j) and IL-6 production (Fig. 3k).

Our analysis also showed that airway cellular infiltration, which represents lung airway inflammation, was suppressed by inhibitors of 5-LOX, 12-LOX, or BLT2 (Fig. 3d, i). Furthermore, we found that treatment with LY255283 effectively decreased the levels of p-IκBα, an indicator of NF-κB activation, in mouse lung tissue (Fig. 3l). These results are consistent with previous results demonstrating that the BLT2 pathway stimulates the production of inflammatory cytokines via the transcription factor NF-κB^32^. Moreover, the LY255283 treatment significantly reduced the levels of inflammatory cytokines and chemokines associated with the progression of lung cancer^33–39^ (IL-1β, TNFα, CXCL1, GM-CSF, MUC4, and MUC5AC; Fig. 3m). Taken together, these results suggest that the BLT2 cascade modulates the synthesis of various proinflammatory agents, including IL-6, that are critical for KRAS-driven lung tumor formation.

### KrasG12D-driven lung tumor formation and lung inflammation are attenuated in *Blt2* KO mice

**Fig. 4:**
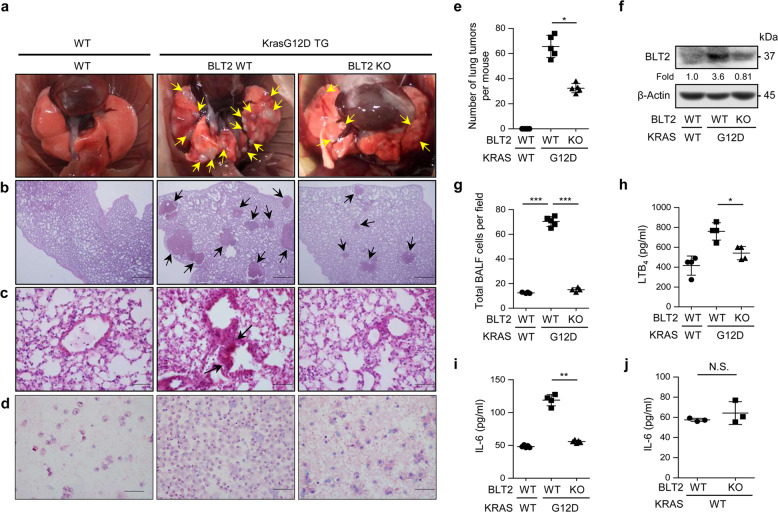
BLT2 knockout suppresses KrasG12D-driven lung tumor formation and IL-6 production. **a** Representative images of lung lesions and **b** H&E staining of lung tissue. Scale bars, 500 μm. **c** Airway cellular infiltration in the indicated experimental groups. The arrows indicate infiltrated cells. Scale bars, 100 μm. **d** Representative images of BALF cytospin preparations isolated from each group. The results are representative of five independent experiments with similar results. **e** The number of lung tumors in the mice was counted. The data are presented as the mean ± SD; *n* = 5. *p < 0.05 according to Student’s *t*-test. **f** BLT2 protein levels in lung lysates as measured by western blotting. The results are representative of three independent experiments with similar results. Band intensities were quantified using ImageJ and are expressed as the fold change relative to the control value. **g** The average number of BALF cells per field of view was determined. The results are presented as the mean ± SD values; *n* = 5. ***p < 0.001 with Student’s *t*-test. Lung tissue extracts were collected to analyze (**h**) LTB_4_ and (**i**, **j**) IL-6 by ELISAs. The data are presented as the mean ± SD; *n* = 5. *p < 0.05 and **p < 0.01 according to Student’s *t*-test. N.S. not significant.

To further determine whether BLT2 is involved in KRAS-driven lung tumor formation, we established a Kras^G12D^/BLT2-KO double-mutant mouse model. The Kras^G12D^/BLT2-KO mice had fewer lung nodules than the Kras^G12D^ mice (Fig. 4a, b, e). The BLT2 expression levels in the lung tissues of these double-mutant mice were lower than those in the lung tissues of the Kras^G12D^ mice (Fig. 4f). Furthermore, lung airway inflammation (Fig. 4c) and the number of immune cells in BALF (Fig. 4d, g) were substantially reduced in the Kras^G12D^/BLT2-KO mice compared with the Kras^G12D^ mice. The levels of LTB_4_ were also reduced in the Kras^G12D^/BLT2-KO mice (Fig. 4h), consistent with the feedback regulatory mechanism between eicosanoid receptors and their ligands^40,41^. IL-6 expression was also reduced in the Kras^G12D^/BLT2-KO mice compared with the Kras^G12D^ mice (Fig. 4i). In addition, the IL-6 level did not differ between the KRAS^WT^/BLT2-KO mice and the WT mice, suggesting that the effect of BLT2 KO is specific to KRAS-induced IL-6 production (Fig. 4j). Together, these results suggest that *Blt2* KO effectively suppresses KrasG12D-driven lung inflammation and tumor formation.

### High BLT2 expression is observed in lung cancer patients with the KrasG12D mutation

**Fig. 5:**
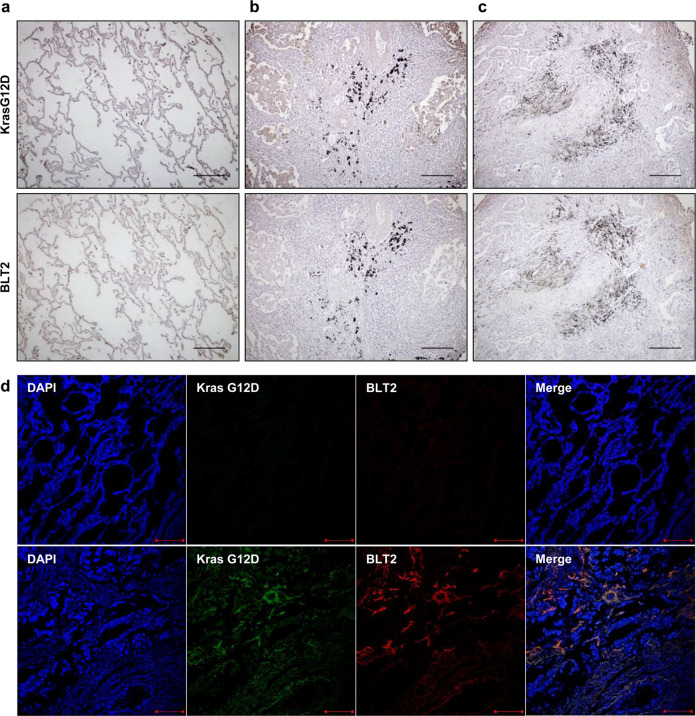
High expression levels of BLT2 in lung adenocarcinoma patients with KRAS mutations. **a**–**c** IHC analysis of KrasG12D and BLT2 expression in lung adenocarcinoma patients (**a** normal lung tissue; **b**, **c** lung adenocarcinoma tissue). Scale bars, 400 μm. **d** IF analysis of KrasG12D and BLT2 expression in lung adenocarcinoma patients (upper panel, normal lung tissue; lower panel, lung adenocarcinoma tissue). Scale bars, 100 μm.

**Fig. 6:**
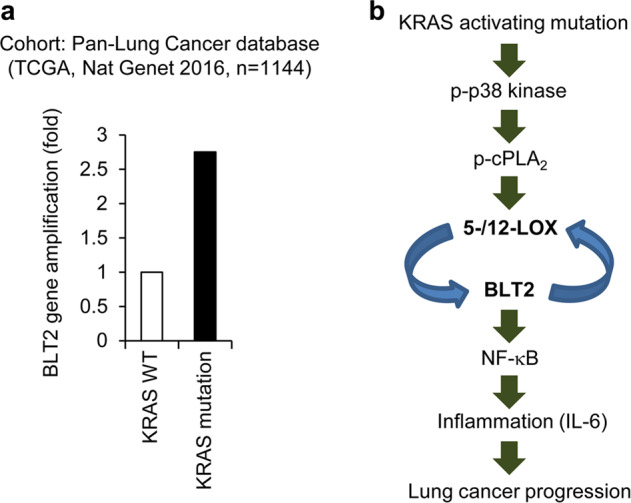
KRAS-mutant lung cancer patients show elevated BLT2 gene amplification. **a** Fold increased BLT2 gene amplification in KRAS-mutant lung cancer patients compared with KRAS WT lung cancer patients. The Pan-Lung Cancer database (TCGA, Nat Genet 2016) cohort of 1144 lung cancer patients was analyzed by cBioPortal. **b** A model summarizing the findings from our study.

Next, we further examined the link between BLT2 expression and KrasG12D status in lung cancer patients. Thus, to determine whether BLT2 expression is increased in patients with KrasG12D-expressing lung cancer, we performed IHC staining of paraffin-embedded tissue sections derived from 45 patients with malignant lung adenocarcinoma. The expression of KrasG12D and BLT2 was not detected in normal human lung tissue (Fig. 5a). In contrast, the lung adenocarcinoma tissue sections showed positive staining for KrasG12D, and the KrasG12D-stained samples showed high BLT2 expression in the KrasG12D-positive region, consistent with the suggested role of elevated BLT2 expression in KRAS-driven lung cancer (Fig. 5b, c). To further analyze the role of elevated BLT2 expression in KRAS-driven lung cancer, we performed a co-staining experiment in lung tissues by IF analysis. As shown in Fig. 5d, we observed a clear colocalization of KrasG12D and BLT2 expression (Fig. 5d). Moreover, to further demonstrate the linkage between BLT2 expression and KRAS mutation in human lung cancer, we analyzed the patient database stratified by KRAS mutation. We used the Pan-Lung Cancer (TCGA, Nat Genet 2016, *n* = 1144) database to analyze the specificity of KRAS mutation and BLT2. We found that the KRAS-mutant lung cancer patients showed 2.7-fold increased BLT2 gene amplification compared with the KRAS WT lung cancer patients (Fig. 6a). Together, our results indicate that BLT2 has a potential role in KRAS-driven lung cancer (the proposed signaling model is summarized in Fig. 6b) and that inhibition of the BLT2 cascade may be a new therapeutic strategy against KRAS-driven lung cancer.

## Discussion

In the present study, we demonstrated that the BLT2 cascade is critical for the progression of KRAS-driven lung cancer. Treatment with BLT2 cascade inhibitors clearly suppressed IL-6 production and lung tumor nodule formation in a KrasG12D-driven lung cancer mouse model. The contributory role of BLT2 in the proliferation of KRAS-driven lung tumors was further demonstrated in the Kras^G12D^/BLT2 KO model. These double-mutant mice showed clear reductions in the level of IL-6 and the number of lung nodules compared with the Kras^G12D^/BLT2 WT mice. In support of the contributory role of BLT2 in KRAS-driven lung cancer progression, IHC analysis of tissues obtained from patients with KrasG12D mutant malignant lung adenocarcinoma showed high levels of BLT2 expression.

Lung cancer is currently considered a difficult-to-treat disease. More than half of lung cancer patients have KRAS or EGFR mutations^42^; however, there is no available effective cancer therapy for patients with KRAS-mutant lung cancer, in contrast to those with EGFR-mutant lung cancer. Patients with EGFR-mutant lung cancer are commonly sensitive to receptor tyrosine kinase inhibitor (RTKi) drugs, which show some ability to improve overall survival^43,44^. However, patients with KRAS-mutant lung cancer are not sensitive to RTKi drugs; thus, an effective alternative treatment is urgently needed. Mutations in codons 12, 13, and 61 of KRAS have oncogenic potential, and KrasG12X mutations are the most abundant mutation types in lung cancer patients^45^. Among these mutations, KrasG12D is found in ~15% of KRAS-mutant lung cancer cases, and more than half of never-smoking lung cancer patients have the KrasG12D mutation^46^. Based on these clinical observations, we used a mouse model with a lung-specific expression of the KrasG12D mutation to test BLT2 as an alternative target for KRAS-driven lung cancer. In this lung cancer model, inhibition of the BLT2 cascade effectively attenuated lung tumor progression.

A link between the KrasG12D mutation and BLT2 expression in lung cancer was also suggested by IHC and IF analysis of tissues derived from patients with malignant lung adenocarcinoma (Fig. 5). These KrasG12D-positive samples showed highly elevated BLT2 expression, in agreement with the contributory role of BLT2 in KRAS-driven human lung cancer. Of course, further experiments with more KRAS mutant lung cancer patient samples are needed to demonstrate the linkage between BLT2 expression and KRAS mutation in human lung cancer. The mechanism by which the expression levels of BLT2 and its ligand-producing enzymes (5-LOX and 12-LOX) were highly increased by mutant KRAS is not clear, but we speculate that p38 kinase is involved. Previous studies have demonstrated that p38 kinase stimulates the phosphorylation of cPLA_2_, thus activating the cPLA_2_-5-/12-LOX cascade^47,48^. Indeed, we observed that the KrasG12D mutant stimulated p38 kinase activity in the lung tissues of a KRAS-driven lung cancer mouse model (Supplementary Fig. 2a). In addition, the protein expression levels of 5-/12-LOX and BLT2 in SK-LU-1 cells were diminished by treatment with a p38 inhibitor (Supplementary Fig. 2b).

Inflammatory signaling plays an important role in cancer progression. In particular, inflammatory lipid mediators such as eicosanoids and their receptors have been suggested to establish a tumor-friendly environment by acting as local mediators. For example, cyclooxygenase (COX)-2 belongs to the proinflammatory eicosanoid family and increases lung cancer progression and lung inflammation^49,50^. In contrast, the roles of LOX- or LOX-derived lipid mediators in lung cancer, especially KRAS-driven lung cancer, remain to be elucidated. In this study, we demonstrated that the 5-/12-LOX-BLT2 cascade is highly amplified during KRAS-driven lung cancer progression (Fig. 2c). The expression level of BLT2 was highly elevated in KRAS-mutant lung cancer cells and in both mouse and human lung cancers with KrasG12D mutations. We speculate that LTB_4_, a product of 5-LOX, or 12(*S*)-HETE, a product of 12-LOX, interacts with its receptor, BLT2, to establish or mediate the tumor-promoting environment^51^. In addition, we found that the inhibition of BLT2 reduces the levels of its ligand-producing enzymes and ligands (Fig. 4h and Supplementary Fig. 3), and this feedback regulation is frequently observed in eicosanoid receptors^40,41^. In this tumor-friendly environment, BLT2-mediated autocrine positive feedback loops could lead to more aggressive amplification of tumor progression^52–54^. In conclusion, to our knowledge, this report is the first to define the role of BLT2 in KRAS-driven lung cancer. We anticipate that these new findings will facilitate the development of strategies against lung cancer, especially KRAS-mutant lung cancer.

## Supplementary information

Supplementary Information
