# Supplementary material for: Leukotriene B4 receptor-2 contributes to KRAS-driven lung tumor formation by promoting interleukin-6-mediated inflammation
Source: Exp Mol Med. 2021 Oct 11;53(10):1559–68. doi: 10.1038/s12276-021-00682-z (PMC8569214; doi:10.1038/s12276-021-00682-z)
Supplement: Supplementary file 1 — Supplementary Information [file 12276_2021_682_MOESM1_ESM.pdf]

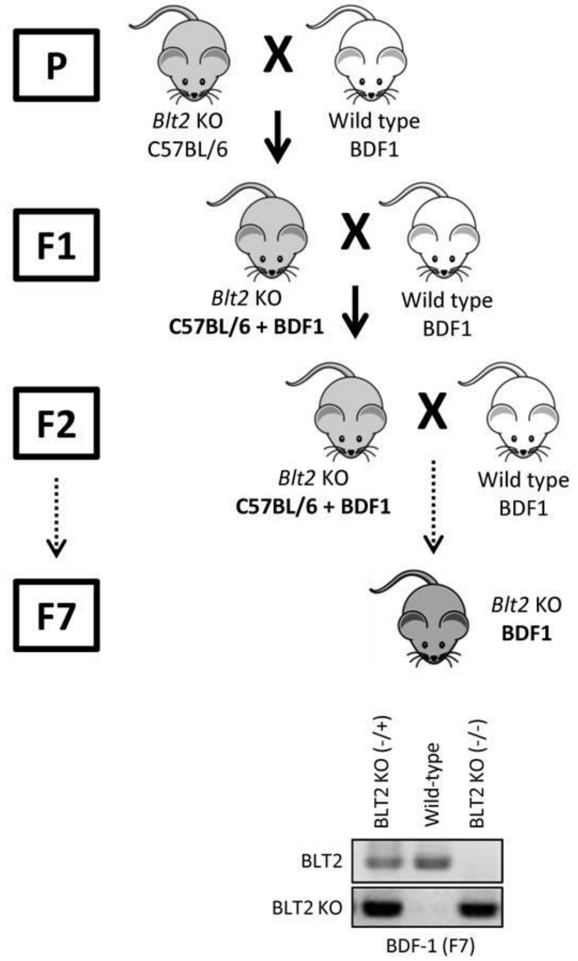

Supplementary Figure 1. Scheme for the generation of Kras^{G12D}/BLT2 knockout mice and genotyping of F7 mice.

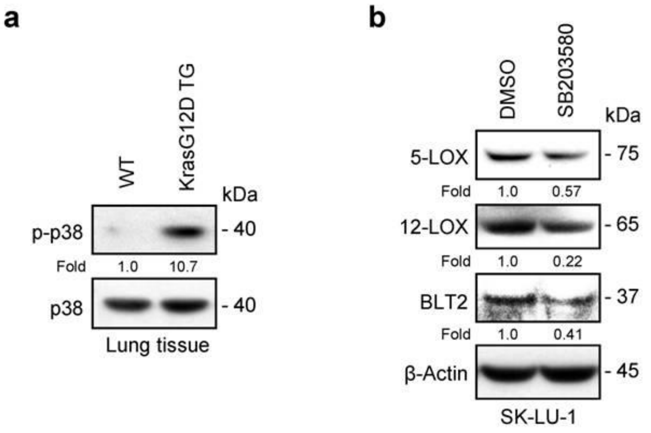

Supplementary Figure 2. p38 kinase as upstream of the BLT2 cascade. a Western blot analysis of p-p38 expression in mouse lung tissue lysates. Three mice were tested for the analysis. Similar results were obtained and shown are the representative data. **b** Western blot analysis of 5-/12-LOX and BLT2 protein expression after treatment of SK-LU-1 cells with 10 μM SB203580 (p-p38 inhibitor). The western blot data are representative of three independent experiments with similar results.

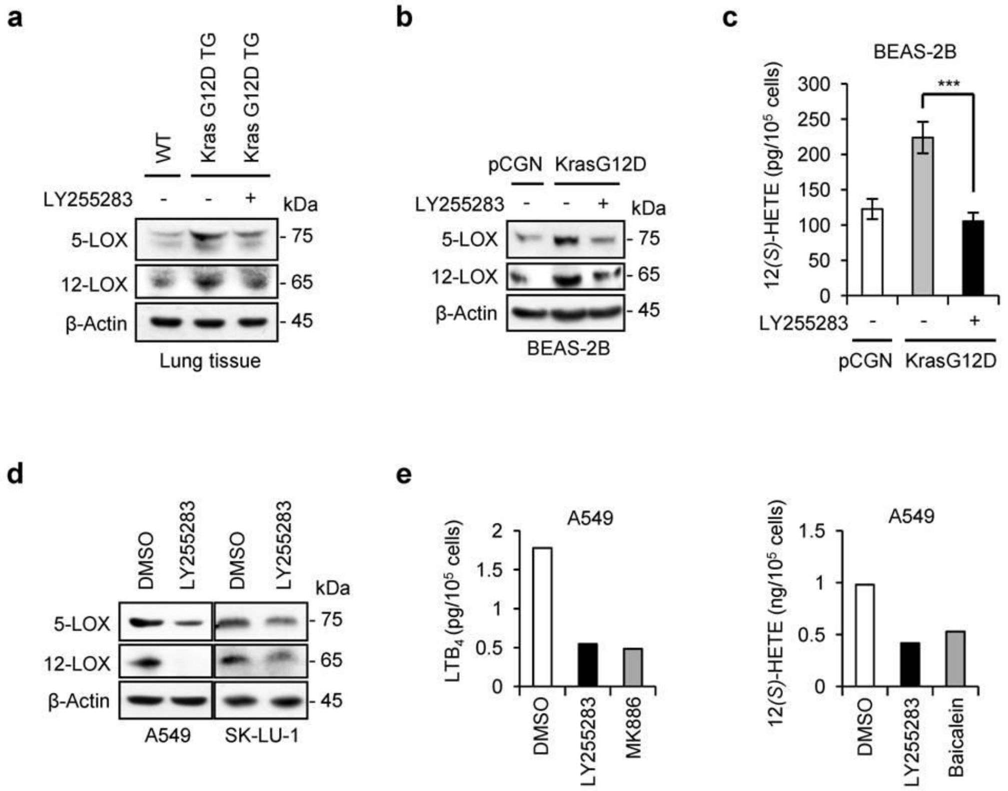

Supplementary Figure 3. Reduced production of BLT2 ligands by the inhibition of BLT2. **a** Western blot analysis of mouse lung tissue lysates was performed to assess the levels of 5-/12-LOX. Three mice were tested for the analysis. Similar results were obtained and shown are the representative data. **b** 5-/12-LOX protein expression was assessed by western blot. BEAS-2B cells transiently transfected with the KrasG12D vector or the pCGN control vector were treated with 10 µM LY255283 for 12 h, and cell lysates were analyzed by western blotting. The western blot data are representative of three independent experiments with similar results. **c** The level of 12(*S*)-HETE in the cell culture supernatant was measured by ELISA. ELISA data are presented as the mean ± SD of three independent experiments. The data were analyzed using an unpaired two-tailed Student's t-test. ***$p < 0.001$ **d** Western blot analysis of 5-/12-LOX protein expression after treatment of A549 or SK-LU-1 cells with 10 µM of LY255283. The western blot data are representative of three independent experiments with similar results. **e** A549 cells were treated with the indicated inhibitors for 12 h, and the LTB_4 and 12(*S*)-HETE concentrations in the culture supernatants were measured by ELISA.

Supplementary Table 1. Primer sequences used in the study

BLT2 forward primer<br>BLT2 reverse primer	AGC CTG GAG ACT CTG ACC GCT TTC G<br>GAC GTA GAG CAC CGG GTT GAC GCT A
Human IL-6 forward primer<br>Human IL-6 reverse primer	CCA GTA CCC CCA GGA GAA GA<br>GCA TCC ATC TTT TTC AGC CA
Human KRAS forward primer<br>Human KRAS reverse primer	TAC AGT GCA ATG AGG GAC CA<br>AGG CAT CAT CAA CAC CCT GT
Mouse genotyping BLT2 forward primer<br>Mouse genotyping BLT2 reverse primer	CAG CAT GTA CGC CAG CGT GC<br>CGA TGG CGC TCA CCA GAC G
Mouse genotyping KrasG12D forward primer<br>Mouse genotyping KrasG12D reverse primer	CAG GAA CAA ACA GGC TTC AAA<br>TTA TGG CAA ATA CAA AAG AAA GC
Mouse genotyping BLT2 KO forward primer<br>Mouse genotyping BLT2 KO reverse primer	AGC TTG AGA GAG CTT CAG TAC ACT CC<br>GCA GCC TCT GTT CCA CAT ACA CTT CA
Mouse IL-1β forward primer<br>Mouse IL-1β forward primer	GAA AGC TCT CCA CCT CAA TG<br>GCC GTC TTT CAT TAC ACA GG
Mouse TNF-α forward primer<br>Mouse TNF-α forward primer	ATG AGC ACA GAA AGC ATG ATC<br>TAC AGG CTT GTC ACT CGA ATT
Mouse CXCL1 forward primer<br>Mouse CXCL1 forward primer	CTT GAA GGT GTT GCC CTC AG<br>TGG GGA CAC CTT TTA GCA TC
Mouse GM-CSF forward primer<br>Mouse GM-CSF forward primer	GTG GTC TAC AGC CTC TCA GCA C<br>GGG GGC AGT ATG TCT GGT AG
Mouse MUC4 forward primer<br>Mouse MUC4 forward primer	TGA TCC TGC CTA TAC TGC CC<br>GGA CGG TAC TTC TCC ATT GC
Mouse MUC5AC forward primer<br>Mouse MUC5AC forward primer	CCA TGC AGA GTC CTC AGA ACA<br>GGC CTC TCC TAC CTC CAA GA
GAPDH forward primer<br>GAPDH reverse primer	CTG CAC CAC CAA CTG CTT AGC<br>CTT CAC CAC CTT CTT GAT GTC
